# Dosimetric review of cardiac implantable electronic device patients receiving radiotherapy

**DOI:** 10.1120/jacmp.v16i1.5189

**Published:** 2015-01-08

**Authors:** Joann I. Prisciandaro, Akash Makkar, Colleen J. Fox, James A. Hayman, Laura Horwood, Frank Pelosi, Jean M. Moran

**Affiliations:** ^1^ Department of Radiation Oncology University of Michigan Ann Arbor MI; ^2^ Department of Internal Medicine University of Michigan Ann Arbor MI; ^3^ Arizona Heart Rhythm Center Phoenix AZ; ^4^ Department of Radiation Oncology Dartmouth‐Hitchcock Medical Center Lebanon NH

**Keywords:** cardiac implantable electronic device, pacemaker, defibrillator, radiation therapy

## Abstract

A formal communication process was established and evaluated for the management of patients with cardiac implantable electronic devices (CIEDs) receiving radiation therapy (RT). Methods to estimate dose to the CIED were evaluated for their appropriateness in the management of these patients. A retrospective, institutional review board (IRB) approved study of 69 patients with CIEDs treated with RT between 2005 and 2011 was performed. The treatment sites, techniques, and the estimated doses to the CIEDs were analyzed and compared to estimates from published peripheral dose (PD) data and three treatment planning systems (TPSs) — UMPlan, Eclipse's AAA and Acuros algorithms. When measurements were indicated, radiation doses to the CIEDs ranged from 0.01–5.06 Gy. Total peripheral dose estimates based on publications differed from TLD measurements by an average of 0.94 Gy (0.05–4.49 Gy) and 0.51 Gy (0–2.74 Gy) for CIEDs within 2.5 cm and between 2.5 and 10 cm of the treatment field edge, respectively. Total peripheral dose estimates based on three TPSs differed from measurements by an average of 0.69 Gy (0.02–3.72 Gy) for CIEDs within 2.5 cm of the field edge. Of the 69 patients evaluated in this study, only two with defibrillators experienced a partial reset of their device during treatment. Based on this study, few CIED‐related events were observed during RT. The only noted correlation with treatment parameters for these two events was beam energy, as both patients were treated with high‐energy photon beams (16 MV). Differences in estimated and measured CIED doses were observed when using published PD data and TPS calculations. As such, we continue to follow conservative guidelines and measure CIED doses when the device is within 10 cm of the field or the estimated dose is greater than 2 Gy for pacemakers or 1 Gy for defibrillators.

PACS number: 87.55.N‐

## I. INTRODUCTION

There has been a steady increase in the number of patients presenting with cardiac implantable electronic devices (CIEDs) for radiation therapy (RT), namely implantable cardiac pacemakers (ICPs) and cardioverter defibrillators (ICDs).^1^, ^2^ These devices have undergone significant technological improvements over the last few decades, resulting in lower power consumption, greater reliability, and prolonged generator lifespan.^(3)^ However, the complementary metal oxide semiconductors (CMOS) utilized in their circuits are known to be susceptible to ionizing and electromagnetic radiation, which may result in transient or permanent device defects.^4^, ^5^, ^6^, ^7^, ^8^, ^9^, ^10^, ^11^ Recommendations from CIED manufacturers vary widely. Some CIED manufacturers provide total dose and dose rate recommendations, while others state that no safe radiation dosage can be specified. As such, radiation oncology facilities have relied on consensus‐based management guidelines which suggest that the CIED be kept outside the primary radiation field(s).^7^, ^10^, ^11^

AAPM Task Group 34^(10)^ provided six recommendations for managing patients with pacemakers, three of which are still relevant today: 1) the ICP should not be irradiated with primary radiation; 2) the dose to the ICP should be estimated prior to RT; and 3) if the cumulative dose to the ICP is expected to exceed 2 Gy, the ICP should be interrogated prior to RT and weekly during treatment. Several of these recommendations are still followed today, even though RT delivery techniques have changed. Some have argued that the recommended cumulative dose limit may be too conservative and/or that other factors, such as the patient's device dependency, should play a role in the perceived potential risk of RT.^7^, ^11^, ^12^ However, for patient safety, most guidance documents continue to recommend that the CIED dose be estimated and/or measured to determine if treatment planning modifications are necessary and to determine the appropriate level of patient monitoring.

Typically, CIEDs are located at the periphery of the treatment field(s) where clinical dose calculation algorithms are less accurate.^13^, ^14^, ^15^, ^16^, ^17^ Peripheral dose (PD) is made up of contributions from leakage radiation, collimator head scatter, and internal scatter.^15^, ^16^, ^18^ The contributions of each of these components vary based on the distance from the field edge^(15)^ and the treatment technique. Additionally, the dosimetric accuracy of TPSs are known to decrease with distance from the treatment field aperture(s).^19^, ^20^ Since TPSs are not commissioned for PD calculations and their accuracy is known to decrease with increasing distance from the field edge,^(19)^ published data are often used to estimate PDs.^14^, ^15^, ^16^, ^17^, ^18^, ^21^ Regardless of the technique utilized to assess dose to CIEDs, the method's accuracy should be high enough for the determination of the potential risk the treatment may pose.^(7)^

A retrospective, IRB‐approved study for patients with CIEDs treated with RT between 2005 and 2011 was conducted at the University of Michigan as a collaborative effort between the Department of Radiation Oncology and the Cardiac Electrophysiology section. Based on the data collected in this study, a summary of the effects of RT on the function of CIEDs, as well as an established communication protocol between Electrophysiology and Radiation Oncology, was published.^(22)^ The goal of the current study is to investigate dosimetric parameters for CIED patients receiving RT to assess whether a correlation between treatment techniques and CIED events can be identified, and to determine if our measurement protocol continues to be appropriate and necessary.

## II. MATERIALS AND METHODS

Sixty‐nine patients with CIEDs (19 ICDs and 50 ICPs) were treated in the Department of Radiation Oncology between 2005 and 2011. The cardiac devices were manufactured by Biotronik, Boston Scientific, Medtronic, or St. Jude, and patients were treated to a variety of sites, including 21 patients treated to multiple sites.^(22)^ Thirty‐five patients were treated with 3D conformal plans which included at least one wedged field, 13 patients were treated with at least one intensity‐modulated radiation therapy (IMRT) plan (1.8–2.5 Gy/fx), six patients were treated with at least one stereotactic body radiation therapy (SBRT) lung plan (8–18 Gy/fx), and one patient was treated with two intracranial stereotactic radiosurgery (SRS) plans (single 16 and 21 Gy treatment). In addition, 36 patients (24 with ICPs and 12 with ICDs) were treated with a plan that included at least one high‐energy (16 MV) photon beam. A summary of the treatment techniques and the number of treatment plans generated is provided in Table 1. All clinical treatment plans were developed using an in‐house TPS, UMPlan, which is based on principles outlined in Fraass et al.,^(23)^ but modified for improved heterogeneity calculations. Patients were treated on one or more of the following linac models: 2100CD, 21‐EX, 21‐iX, or 600CD (Varian Medical Systems, Palo Alto, CA).

**Table 1:** A summary of the treatment techniques and plans for the 69 patient cohort. The number of plans listed per technique includes the initial treatment plan, replans, and boosts

*Treatment Technique*	*Number of Treatment Plans*	*Dose/Fraction Range (Gy/fx)*	*Total Dose Range (Gy)*
Electron(s)	16	2–4	4–50
3D Conformal	53	1.5–8	4–68.4
3D Conformal with wedge(s)	53	1.5–8	4–64.8
IMRT	17	1.8–2.5	26–77.7
SRS	2	16–21	16–21
SBRT	9	8–18	40–54

The distances from the edge of the radiation field to the CIEDs for this patient cohort ranged from 1.5 cm to greater than 40 cm. Five patients (2 with ICPs and 3 with ICDs) with devices <2.5cm from the treatment field edge were determined to be medically high‐risk and did not undergo CIED relocation. These patients had their CIEDs monitored daily by Electrophysiology while receiving RT. Clinically, the dose to the CIED for each patient was estimated prior to the start of treatment using published PD data (Fraass and van de Geijn^(14)^ for photon beams and Chow and Grigorov^(21)^ for electron beams). Estimates were biased to the most conservative dose based on the available field size, distance, and depth data in the publications. If the estimated dose to the device was expected to exceed 1 Gy for ICDs^(4)^ or 2 Gy for ICPs,^(10)^ or if the device was less than 10 cm from the edge of a treatment field, the dose was measured during the first treatment using TLD‐100 disks (Thermo Fisher Scientific, Oakwood Village, OH). The TLDs were placed under 1.0–2.0 cm of bolus on the CIED generator in the position of nearest approach to the treatment fields and were in place for the imaging (when applicable) and treatment fields. After irradiation, the TLDs were annealed for 10 min in a 100°C preheated oven. The TLD disks were then individually read using a Harshaw model 3500 manual reader (Thermo Fisher Scientific). The total dose for the treatment course was estimated based on this measurement to enable determination of the appropriate level of oversight.

For the purpose of this study, the CIED dose was also estimated using published PD data from Stovall et al.,^(15)^ Stern,^(16)^ and Mutic and Klein^(17)^ for patients whose CIED was less than 10 cm from any field edge. A reference point was added in the TPS at the location of the CIED closest to the field edge. The dose to this point was calculated using three TPSs: 1) the clinical treatment planning algorithm, UMPlan; 2) Eclipse version 11.031 with the analytical anisotropic algorithm (AAA); and 3) Eclipse version 11.031 with the Acuros algorithm (Varian Medical Systems). For the Acuros calculations, the CT calibration curve was extended to include CT data for titanium based on the recommendation of Gossman et al.^(3)^ The CIED generator and leads were contoured and assigned the material properties of titanium (density of 4.42 g/cm^3^). The high‐density artifacts from the CIED generator and leads were segmented and assigned a water‐equivalent density (1 g/cm^3^).

## III. RESULTS

**Figure 1:**
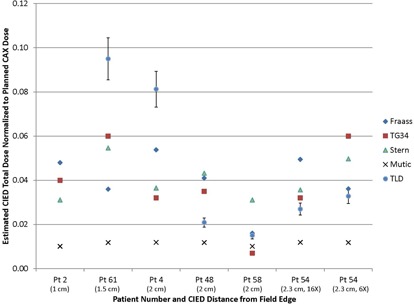
Estimated total CIED dose normalized to the prescribed central axis (CAX) dose for all patients and plans where the CIED was less than 2.5 cm from the edge of a treatment field. The estimated PDs are based on Fraass and van de Geijn,^(14)^ TG34,^(15)^ Stern,^(16)^ and Mutic and Klein,^(17)^ and the estimates for Patient 54 are shown for both 16X and 6X treatment plans that were utilized for this patient (16X plan was discontinued after one treatment fraction).

For this cohort of 69 patients, dose estimates based on TLD measurements ranged from 0.9 to 506 cGy and 4 to 169 cGy for ICPs and ICDs, respectively. Figure 1 and Table 2 present the estimated and measured doses to the CIEDs for devices which were less than 2.5 cm and 2.5–10 cm from the edge of the treatment field, respectively. For this study, the PD estimates were based on data published by Fraass and van de Geijn,^(14)^ Stovall et al.,^(15)^ Stern,^(16)^ and Mutic and Klein^(17)^ for photons and Chow and Grigorov^(21)^ for electrons. For distances of less than 2.5 cm, the total PD estimates based on published data^14^, ^15^, ^16^, ^17^ differed from measurements by an average of 0.94 Gy (0.05–4.49 Gy) across the four publications (Fraass and van de Geijn^(14)^: 0.89 Gy [0.05–3.19 Gy]; TG34^(15)^: 0.78 Gy [0.25–1.89 Gy]; Stern^(16)^: 0.88 Gy [0.36–2.18]; and Mutic and Klein^(17)^: 1.22 Gy [0.25–4.49Gy]). For distances between 2.5 cm and 10 cm, the total PD estimates based on these same publications differed from measurements by 0.51 Gy (0–2.74 Gy) — (Fraass and van de Geijn^(14)^: 0.76Gy [0.02–2.26Gy]; TG34^(15)^: 0.41 Gy [0.01–1.67 Gy]; Stern^(16)^: 0.45 Gy [0.04–2.50 Gy]; and Mutic and Klein^(17)^: 0.45 Gy [0–2.74 Gy]).

Table 3 summarizes the TPS calculated doses to the CIEDs for patients whose device was less than 2.5 cm from the field edge based on the TPSs’ algorithms. When compared to the TLD measurements, the CIED total dose differed by an average of 0.69 Gy (0.02–3.72 Gy) across all three TPSs (UMPlan: 0.54 Gy [0.06–1.61 Gy]; AAA: 0.78 Gy [0.02–3.41 Gy]; Acuros: 0.73 Gy [0.05–3.72 Gy]).

Of the 69 patients evaluated, only two ICD patients experienced a partial reset of their device, which resulted in the loss of the patient's diagnostic data. Both patients were treated with plans that included at least one $16\;\text{MV}\;(\%\text{dd}({10)}_{x}=77.7)$ photon beam. The ICD for one of these patients was <2.5cm from the radiation treatment fields.

**Table 2:** Estimated vs. measured CIED doses for all patients and plans in which the CIED was between 2.5 and 10 cm from the edge of a treatment field (distance between CIED and closest treatment field edge indicated in the column labeled “d”). The estimated doses were based on the publications of Fraass and van de Geijn,^(14)^ TG34,^(15)^ Stern,^(16)^ and Mutic and Klein,^(17)^ and Chow and Grigorov,^(21)^ as well as *in vivo* measurements

	*Plan Parameters*						*Estimated Total Dose*	
*Pt. #*	*Treatment Site*	*Total Prescription Dose (Gy) (Dose/fx [Gy/fx])*	*E (MV or MeV)*	*Treatment Technique*	*Device*	*d (cm)*	*Dose (Gy) Fraass/TG34/Stern/Mutic*	*TLD Result (Gy)*
4	Upper extremity	8 (8)	6X	3D with wedge	ICD	4	0.43/ 0.40/ 0.21/ 0.09	0.49
8	Esophagus	50 (2)	6X	3D	ICP	5	2.75/ 2.00/ 1.28/ 0.58	0.58
11	Esophagus	30 (1.5)	16X	3D	ICD	3	1.59/ 0.30/ 1.46/ 0.33	0.72
	Esophagus	30 (1.5)	16X	3D	ICD	4.5	1.59/ 0.30/ 0.58/ 0.33	0.54
	Esophagus	15 (1.5)	16X	3D with wedge	ICD	5	0.80/ 0.15/ 0.22/ 0.16	—
14	Breast and thorax	39 (3)	6X	3D with wedge	ICP	4	1.01/ 0.86/ 0.69/ 0.42	0.73
16	Head and neck	60 (2)	6X	IMRT	ICP	5	3.24/ 0.66/ 1.33/ 0.69	1.02
17	Head and neck	50 (2.5)	6X	3D with wedge	ICP	5	2.70/ 1.00/ 0.74/ 0.54	0.44
20	Breast and thorax	60 (2)	6X, 9e,12e	3D	ICP	9.9	0.96/ 0.60/ 0.84/ 0.60	0.75
27	Breast and thorax	37.5 (2.5)	6X	3D with wedge	ICP	5	0.56/ 0.75/ 0.56/ 0.38	0.36
31	Esophagus	50 (2)	16X	3D	ICP	8	0.95/ 0.25/ 0.37/ 0.50	0.79
33	Head and neck	70 (2)	6X	IMRT	ICP	5	3.85/ 2.66/ 1.68/ 0.81	2.84
35	Breast and thorax	60 (2)	6X	3D	ICP	4	3.30/ 2.88/ 1.68/ 0.71	3.45
36	Head and neck	16 (2)	16X	3D with wedge	ICP	3.5	2.50/ 0.51/ 0.69/ 0.54	0.89
38	Breast and thorax	30 (3)	6X	3D	ICP	5.5	1.62/ 0.39/ 0.55/ 0.31	0.46
40	Breast and thorax	45 (9)	6X,16X	SBRT	ICP	8	0.50/ 0.18/ 0.15/ 0.32	0.19
41	Head and neck	24 (2)	6X	3D	ICP	9.6	0.13/ 0.12/ 0.12/ 0.19	0.84
42	Breast and thorax	50 (10)	6X,16X	SBRT	ICP	7	0.88/ 0.20/ 0.12/ 0.50	0.50
44	Breast and thorax	50 (10)	6X	SBRT	ICP	3.5	0.75/ 0.53/ 1.58/ 0.54	—
	Breast and thorax	50 (10)	6X	SBRT	ICP	10	0.75/ 0.25/ 0.28/ 0.40	—
45	Head and neck	70 (2)	6X	IMRT	ICD	3	3.50/ 3.36/ 4.19/ 0.78	1.69
47	Upper extremity	50.4 (1.8)	6X	3D	ICD	9.8	1.12/ 0.60/ 0.99/ 0.54	0.70
50	Breast and thorax	24 (2)	6X	3D with wedge	ICP	7	0.86/ 0.26/ 0.22/ 0.24	0.33
55	Head and neck	4 (2)	6X	3D with wedge	ICP	10	0.06/ 0.04/ 0.05/ 0.04	0.09
57	Breast and thorax	50 (2)	16X	3D with wedge	ICP	2.5	2.50/ 0.51/ 1.70/ 0.51	1.58
	Breast and thorax	70 (2)	6X	IMRT	ICP	10	0.39/ 0.34/ 0.42/ 0.56	—
61	Head and neck	6 (2)	6X	3D with wedge	ICP	4.5	0.18/ 0.16/ 0.10/ 0.06	0.57
64	Esophagus	50.4 (1.8)	16X	3D with wedge	ICP	6	1.26/ 0.50/ 0.71/ 0.51	0.86
68	Spine	30 (3)	16X	3D	ICP	10	0.60/ 0.12/ 0.11/ 0.21	0.35
	Spine	20 (4)	6X	3D	ICP	6	0.30/ 0.22/ 0.32/ 0.20	0.12
	*Plan Parameters*	*Estimated Total Dose*						
*Pt. #*	*Treatment Site*	*Total Prescription Dose (Gy) (Dose/fx [Gy/fx])*	*E (MV or MeV)*	*Treatment Technique*	*Device*	*d (cm)*	*Dose (Gy) Chow*	*TLD Result (Gy)*
56	Head and neck	50 (2.5)	6e	Electron	ICD	10	0.2	—

**Table 3:** Comparison of the estimated total CIED dose based on measurements as compared to point doses calculated using three TPS (UMPlan, Eclipse's AAA and Acuros), where “d” represents the distance between CIED and closest treatment field edge

*Plan Parameters*							*Estimated Total Dose to CIED*		*Estimated Total Point Dose to the CIED*		
*Pt. #*	*Treatment Site*	*Delivered Dose (Gy)*	*E (MV)*	*Treatment Technique*	*Device*	*d (cm)*	*TLD Result (Gy)*	*Grid Size (mm)*	*UMPlan (Gy)*	*Eclipse‐AAA (Gy)*	*Eclipse‐Acruos (Gy)*
2	Breast & Thorax	45	6x/16x	3D w/ wedges	ICD	1	N/A	3.0	1.79	1.07	2.90
61	Head & Neck	54	6x	IMRT	ICP	1.5	5.13	3.0	3.52	1.72	1.41
4	Spine	8	6x/16x	3D no wedges	ICD	2	0.65	5.0	0.51	0.38	0.70
48	Breast & Thorax	30	6x/16x	3D no wedges	ICD	2	0.63	3.0	0.56	0.13	0.52
58	Breast & Thorax	50	6x/16x	SBRT	ICP	2	0.75	3.0	1.81	0.54	0.57
54	Esophagus	1.8	16x	3D w/ wedges	ICD	2.3	1.36	3.0	1.30	1.34	1.25
		48.6	6x	3D w/ wedges		2.3	1.59	3.0	1.92	1.87	1.83

## IV. DISCUSSION

This study evaluated patients treated with modern RT techniques for which there is limited information available regarding the sensitivity of these devices to radiation. Several studies^8^, ^24^ suggest a small risk of oversensing to CIEDs from CT irradiation which may result in inappropriate inhibition of pacing output for ICPs, with the additional complication of possibly triggering unneeded tachyarrhythmia therapy in ICDs.^(11)^ These effects were transient and only observed during direct irradiation of the CIED generator.^8^, ^24^ As such, we have instituted a preferred workflow of assessing the patient's device dependency prior to their CT simulation, especially for patients who will receive a dynamic CT scan (e.g., 4D CT) which involves multiple scans of the same region at different time points. For device‐dependent patients, it is important to determine if Electrophysiology should be on‐set during CT simulations.

**Figure 2:**
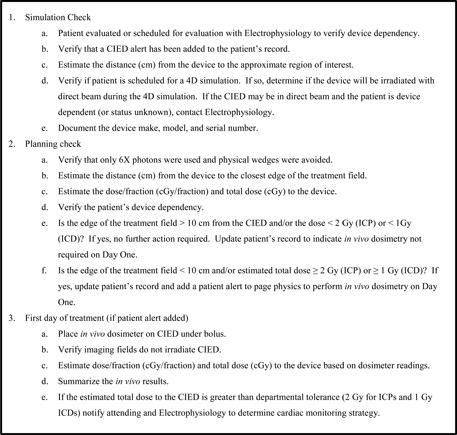
The Radiation Oncology checklist that is completed for CIED patients presenting for RT treatments.

Our workflow includes the medical physicist consulting with the dosimetrist to ensure that planning choices minimize risk to the device by avoiding high‐energy photon beams (>10MV) and physical wedges, unless their absence would significantly compromise the dose distribution. High‐energy photons (>10MV) are nominally avoided because of the increased probability that neutrons will be produced when the photons interact with the atomic nuclei of material they are penetrating. Several studies have suggested that neutrons may cause upsets in the memory or logic circuits of CIEDs.^24^, ^25^ Physical wedges are also avoided to minimize the enhancement of dose due to scatter radiation. Where a wedged dose distribution is desired, dynamic wedges, multiple field segments, or IMRT should be considered. If a patient's CIED is less than 10 cm from the target, the dose to the CIED is estimated prior to the start of treatment via calculation and then with an *in vivo* measurement on the first day of treatment. We follow the checklist in Fig. 2, with the treatment team determining the best approach.

Care must be taken when selecting the appropriate dosimeter to use for these measurements. Thermoluminescent dosimeters have been used widely in clinics for *in vivo* measurements; however, their response may vary based on photon energy and dosimeter model. For instance, TLD‐100 dosimeters have been shown to be more accurate for photon energies of ≤10MV, because they overrespond to thermal neutrons.^(26)^ On the other hand, the TLD‐700 dosimeter has been reported to be insensitive to neutrons, but is not as widely used in clinic. Chan et al.^(27)^ reported a change in sensitivity of 3% (1%–11%) for the TLD‐700, a decrease of 10% (5%–21%) for OSLDs, and an increase of 16% (11%–19%) for skin QED diodes for distances up to 15 cm from edge of the treatment field.

While it is convenient to obtain PD estimates using TPSs, this approach is significantly limited due to inaccuracies in the PD region. Treatment planning systems are not normally commissioned for out‐of‐field calculations, and the accuracy of calculations are known to decrease with increasing distance from the field edge.^19^, ^20^ For Eclipse's AAA algorithm version 8.6 (Varian Medical Systems), Howell et al.^(19)^ observed that PDs were underestimated by the TPS by an average of 40% over a range of 3.75 to 11.25 cm from the field edge, and upwards of 55% at a distance of 11.25 cm from the field edge. Similarly, Huang et al.^(20)^ evaluated PDs for IMRT treatment plans generated using Pinnacle version 9.0.and reported they were underestimated by the TPS by an average of 50%. At distances close to the field edge (3–4 cm), errors in excess of 30% were observed, and at large distances from the treatment field, errors approached 100%.^(20)^

The PD estimates were evaluated by comparing TLD measurements performed on the first treatment day for a given plan with 1) dose estimates based on published PD data, and 2) dose estimates based on TPS calculations. For distances of less than 2.5 cm, the total PD estimates based on published data^14^, ^15^, ^16^, ^17^ differed from *in vivo* measurements by an average of 0.69 Gy using data from four publications. For distances between 2.5 cm and 10 cm, the total PD estimates based on these same publications differed from *in vivo* measurements by an average of 0.51 Gy. These differences may be attributed to uncertainties related to the positioning of the TLD, exclusion of the imaging dose in prediction, limited field sizes, energies, and distances in the published PD data, and differences in the design of the collimator head between the machines in which the published measurements were acquired as compared to the treating linacs. For instance, Fraass and van de Geijn^(14)^ and Stovall et al.^(15)^ performed measurements on teletherapy units and several different linac models prior to the availability of MLCs. Since PD is due to contributions from internal scatter, collimator scatter, and leakage, the presence of an MLC will affect the contribution from collimator scatter. Based on the works of Mutic and Klein^(17)^ and Stern,^(16)^ the presence of a tertiary MLC was shown to reduce PDs. The Mutic study included measurements at 6 and 18 MV for field sizes ranging from $10\times 10\;{\text{cm}}^{2}$ to $25\times 25\;{\text{cm}}^{2}$, whereas the Stern study included measurements for $10\times 10\;{\text{cm}}^{2}$ and $26\times 26\;{\text{cm}}^{2}$, requiring one to interpolate between these data points for an approximate PD estimate.

Table 3 summarizes the calculated doses to the CIEDs for patients whose device was less than 2.5 cm from the treatment field edge using three treatment planning algorithms. When compared to the reported TLD measurements, the dose to the CIED differed by an average of 0.69 Gy across all three TPSs. These differences may be attributed to uncertainties related to the positioning of the TLD, as well as inaccuracies in the TPS algorithm.

Based on current department monitoring guidelines for CIED patients (2 Gy for ICP patients and 1 Gy for ICD patients), the observed difference in the estimated total CIED dose would have resulted in a recommended change in patient monitoring in 12 of 69 patients based on published PD data and 1 of 6 patients based on TPS calculations. With a 17% chance of inadvertently identifying patients in an inappropriate risk category, we continue to follow conservative guidelines and measure CIED doses when the device is within 10 cm of the field or the estimated dose is greater than 2 Gy for pacemakers or 1 Gy for defibrillators.

As presented previously,^(22)^ of the 69 patients evaluated in this study, only two ICD patients experienced a partial reset of their device during their treatment. The estimated total dose to the ICDs for both patients was less than 1 Gy. One of the patient's CIED was within 2.5 cm of the edge of the treatment fields (lung), while the second patient's CIED was >10cm for the region of interest (pelvis). While Mouton et al.^(12)^ has suggested a dose rate effect for ICPs, in their study, the ICPs were only irradiated with direct, high‐energy photons, and dose rate and total accumulated dose were not tested independently. What may be a more plausible cause for the observed partial resets in our patient cohort was that both patients were treated with high‐energy (16 MV) photon beams. As indicated earlier, studies have suggested that neutrons may cause upsets in the memory or logic circuits of CIEDs.^24^, ^25^ Once the partial resets were identified, the treatment plans for both patients were modified and only low‐energy photon beams (6 MV) were utilized. Both patients completed their course of RT without further incident. Based on our experience, we have limited beam energies to less than 10 MV for patients with CIEDs.

## V. CONCLUSIONS

We have developed a standard management plan for CIED patients presenting for RT. We base the degree of surveillance on factors such as distance between the device and treatment site, estimated dose to the device, and patient's device dependency. Few CIED‐related events were observed during RT for these patients. The only noted correlation with treatment parameters for these events was beam energy. As such, we modified our management strategy to limit the beam energy used for treating CIED patients.

Differences in estimated and measured CIED doses were observed when using published PD data and TPS calculations. These differences would have resulted in a recommended change in patient monitoring in 17% of patients based on both published PD data and TPS calculations.

Due to inaccuracies in the calculated dose outside the field edge, we manage the uncertainties in dose estimates by following a conservative guideline and measure when the device is within 10 cm of the field or is estimated to receive 2 Gy (ICPs) or 1 Gy (ICDs). Regardless of the technique utilized to estimate PD to the CIED, the accuracy of the technique should be high enough to determine the potential risk RT may pose on the patient.^(7)^

## ACKNOWLEDGMENTS

The authors would like to thank Sunil Agarwal and Morgan Lusk for their assistance in collecting and organizing the cardiac and radiotherapy related data for this study.
